# Role of Chemical Structure of Support in Enhancing the Catalytic Activity of a Single Atom Catalyst Toward NRR: A Computational Study

**DOI:** 10.3389/fchem.2021.733422

**Published:** 2021-09-08

**Authors:** Thillai Govindaraja Senthamaraikannan, Selvaraj Kaliaperumal, Sailaja Krishnamurty

**Affiliations:** ^1^ Department of Environmental Engineering, Chungbuk National University, Cheongju, Korea; ^2^ Nano and Computational Material Lab, Catalysis Division, CSIR-National Chemical Laboratory, Pune, India; ^3^ Physical Chemistry Division, CSIR-National Chemical Laboratory, Pune, India

**Keywords:** N_2_ activation, single metal atom, pristine graphene, defective graphene, BN-functionalized graphene

## Abstract

Using the periodic density functional theory–based methodology, we propose a potential catalytic system for dinitrogen activation, viz., single metal atoms (Mo, Fe, and V) supported on graphene-based sheets. Graphene-based sheets show an excellent potential toward the anchoring of single atoms on them (Mo, Fe, and V) with adsorption energies ranging between 1.048 and 10.893 eV. Factors such as defects and BN doping are noted to enhance the adsorption energies of single metal atoms on the support. The adsorption of a dinitrogen molecule on metal atom–anchored graphene-based supports is seen to be highly favorable, ranging between 0.620 and 2.278 eV. The adsorption is driven through a direct hybridization between the *d* orbitals of the metal atom (Mo, Fe, and V) on the support and the *p* orbital of the molecular nitrogen. Noticeably, BN-doped graphene supporting a single metal atom (Mo, Fe, and V) activates the N_2_ molecule with a red shift in the N–N stretching frequency (1,597 cm^−1^ as compared to 2,330 cm^−1^ in the free N_2_ molecule). This red shift is corroborated by an increase in the N–N bond length (1.23 Å from 1.09 Å) and charge transfer to an N_2_ molecule from the catalyst.

**Graphical Abstract:**
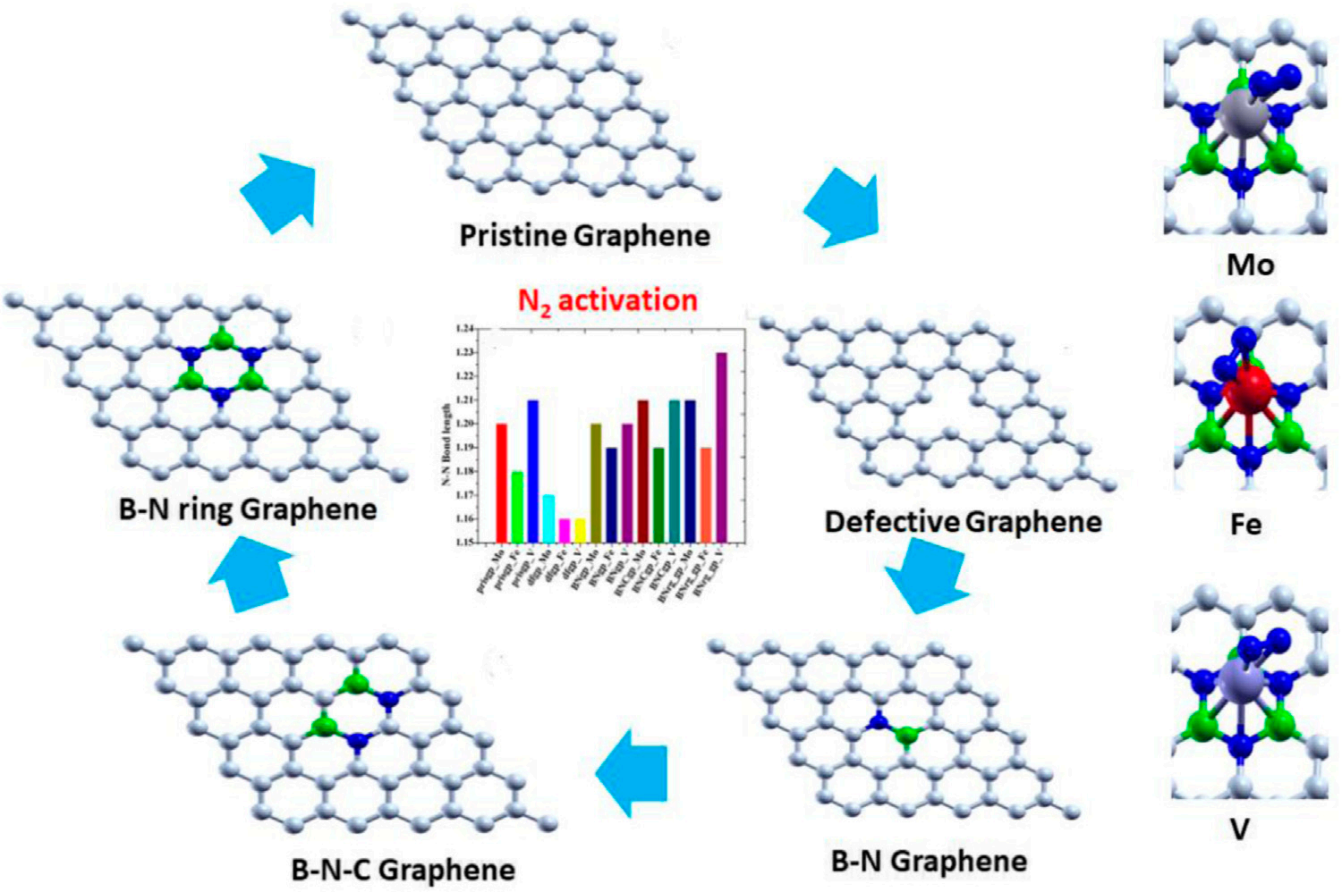
XXX

## Introduction

Ammonia is an important chemical substance for the agriculture, pharmaceuticals, and chemical industries. Natural and synthetic N_2_ fixation is necessary for the existence of all forms of life on Earth. Though the availability of dinitrogen (N_2_) is abundant in air, it requires high energy for fixation and activation owing to its existence of inert triple bonds between nitrogen atoms. Currently, the well-known Haber–Bosch process invented more than a century ago is used for converting dinitrogen (N_2_) in the atmosphere into NH_3_ in the presence of the iron catalyst at an extreme temperature (500°C) and pressure (200 atm) (Fryzuk and Johnson, 2000). The energy- and carbon-intensive Haber–Bosch process consumes 1–2% global energy and, in addition, produces 3% of global CO_2_ emission (Cherkasov et al., 2015). Nevertheless, N_2_ fixation can occur readily under mild conditions by nitrogenase mechanism, the enzyme secreted from very few prokaryotic organisms (Kim and Rees, 1992; Sellmann and Sutter, 1997; Einsle et al., 2002). Researchers have demonstrated the occurrence of biological N_2_ fixation under reasonable or mild conditions in the presence of nitrogenase enzymes, most preferably at the active sites that are rich in Fe and S and also additionally contain Mo or V atoms (Dance, 2008; Stüeken et al., 2015; Tanabe and Nishibayashi, 2016), yet the through kinetics are still disputed. Consequently, exploring an efficient N_2_ reduction catalyst in ammonia synthesis is the main challenge for the organo-metallic researchers. Naturally, N_2_ fixation and activation require a potential catalytic active center to promote nitrogen reduction reaction, via electrons overlapping between the σ bond of N_2_ and the d orbital of the metal center, and the occupied d orbital overlaps with the empty π* bond of N_2_, resulting in the activation of N_2_ by a π bond back-donation mechanism.

On accounting for the quantum confinement of electrons, metal clusters are widely explored as catalysts. Using experimental and theoretical strategies, researchers have explored N_2_ activation on potential inorganic metal clusters (Seh et al., 2017; Liu et al., 2018; Wang et al., 2018). Significantly, Kerpal et al. (2013) have evaluated dinitrogen (N_2_) activation using infrared multiphoton dissociation (IR-MPD) on neutral Ru clusters. Similarly, Roy et al. (2009) have noticed the red-shifted N–N bond stretching frequency around 810 cm^−1^ on solid Li_n_ (2 < n < 8) clusters, particularly the Li_8_ metal cluster showing an exothermic trend in splitting the N–N bond completely. In the midst of metal clusters for evaluating N_2_ activation reaction, Al clusters play a remarkable role. Previously, Jarrold et al. observed low energy barriers for N_2_ activation on Al_44_ and Al_100_ clusters at high temperatures using concerted experimental and theoretical techniques (Cao et al., 2010). Similarly, in another previous report by this group, N_2_ activation potential was observed to be dependent on the phase and structure of the metal cluster (Cao et al., 2009). During the course of N_2_ activation mechanism, conformations with high energy display low energy potential toward the activation of the N_2_ molecule (Kulkarni et al., 2011). Nevertheless, excited state conformations are meta-stable in nature and are notably present only at some characteristic finite temperatures. Hence, there is an obvious demand for more reliable and stable ground state conformations for N_2_ activation. Consequently, heteroatoms such as silicon and phosphorus doped on aluminum clusters appear to be a possible alternative and have better activation than their pristine aluminum clusters (Das et al., 2014).

Moreover, an alternative and experimentally supported route is to enhance the activity of metal-based catalysts by anchoring metal centers on 2D material supports such as graphene and BN, which offers a substantial support to the metal centers to adsorb and activate the N_2_ molecule. Moreover, specific activity per metal atom increases by downsizing the metals from nanoparticles to nanocrystals or hetero-nano framework (Yang et al., 2013; Chen et al., 2014). Single atom catalysts (SACs) have gained more attention in downsizing metals considerably and exhibit the potential of well-dispersed active single atom sites available for atomic utilization (Qiao et al., 2011). Based on these circumstances, SACs exhibiting unique activity with high density of active sites supported on 2D materials can make use of electron sharing for the activation of the inert dinitrogen molecule. A single transition-metal atom or atom clusters supported on N-doped graphene show good nitrogen reduction reaction (NRR) activity (Choi et al., 2015; Li et al., 2016; Fajardo and Peters, 2017; Fei et al., 2018; Yan et al., 2019). Systems such as BiOBr nanosheets, boron anti-sites on BN nanotubes, and Mo-doped boron nitride (BN) have also been reported to have high N_2_ fixation potential (Li et al., 2015; Kumar and Subramanian, 2017; Zhao and Chen, 2017; Légaré et al., 2018).

In the midst of 2D materials, graphene-based supports attract enormous attention in numerous reactions such as water splitting, Guo et al. (2018), and hydrogen evolution reaction (HER) Ouyang et al. (2018). Few experimental groups reported N_2_ fixation using a graphene-based catalytic support (Jeon et al., 2013; Lu et al., 2016; Yan et al., 2018). Several computational investigations have also been explored using graphene-based nanomaterials for N_2_ fixation to compare with the experimental findings. Le et al. reported that the Mo/N-doped graphene-based support dissociates the N_2_ molecule using the density functional theory (DFT) methodology (Le et al., 2014). In a similar approach, Li et al. observed an N_2_ molecule activation to nearly 2.5 Å by fixing the FeN_3_ molecule on a graphene support, in which nitrogen atoms are used as anchoring elements, while iron does the activation job in the FeN_3_ molecule (Li et al., 2016). Kumar et al. (2016) reported N_2_ activation using aluminum clusters doped on the BN-doped graphene support. The rare ability of certain transition complexes to bind to N_2,_ which is attributed to their advantageous combination of unoccupied and occupied d-orbitals that have appropriate energy and symmetry to synergistically accept/back-donate electron density from/to N_2,_ can thus be contrived by giving the appropriate environment to a p-block element. In short, activation of N_2_ is performed by exploiting the electron reservoir property of 2D graphene-based materials. Recently, in our previous investigations, we identified the most active and recyclable SAC/B-graphene composite as the catalyst for NRR activity (Maibam et al., 2019; Maibam and Krishnamurty, 2021). In the present work, using the density functional theory (DFT)-based methodology, we evaluate the possible dinitrogen activation by single metal atoms (Mo, Fe, and V) supported on graphene-based systems such as pristine graphene, defective graphene, BN-doped graphene, BNC-ring graphene, and BN-ring graphene as support materials.

### Computational Details

We use the Vienna Ab Initio Simulation Package (VASP) (Kresse and Furthmller, 1996) with the PBE functional (Perdew et al., 1996) to perform all the first-principles calculations in the present work. The projected augmented wave (PAW) (Blöchl, 1994) method is employed using an energy cutoff of 520 eV to describe the plane wave basis set. The two-dimensional graphene sheets are simulated using periodic boundary conditions. To avoid the interactions between the different nearest neighboring layers, a vacuum space of 20 Å is created along the *Z*-direction. The 5 × 5 supercell with 50 atoms is used as the graphene surface model, and the optimized C–C bond length in the graphene sheet is 1.42 Å.

Pristine graphene, defective graphene, BN-doped graphene, BNC-ring graphene, and BN-ring graphene are designed surface supports, and the structures are further optimized. The structural optimization of all geometries is carried out using the conjugate gradient method (Payne et al., 1992). The Brillouin zone is sampled by a (2 × 2×1) K-point grid using the Monkhorst–Pack scheme (Monkhorst and Pack, 1976). For density of states (DOS) calculations, Monkhorst and Pack generated a (9 × 9×1) set of K points.

The ground state geometries of single transition-metal clusters (Mo, Fe, and V) are adsorbed on the above-mentioned supports and the complexes optimized. The adsorption energy of Mo, V, and Fe on these supports is calculated as follows:

$${\text{E}}_{\text{ad}}{\;=\text{ E}}_{\left(\text{M}--\text{system}\right)}-{\text{E}}_{\left(\text{M}\right)}-{\text{E}}_{\left(\text{system}\right)}\text{,}$$

where E_(M--system)_ represents the energy of the optimized single transition-metal cluster (Mo, Fe, and V) and the designed surface supports. E_(M)_ and E_(system)_ represent the energy of a single metal and surface support, respectively.

Finally, the N_2_ molecule is adsorbed on these active metal clusters (Mo, Fe, and V) on graphene-based surface supports. A parallel mode of adsorption (both the nitrogen atoms are exposed to the metal) is used as this mode has been found to be more effective as compared to the vertical mode. In the vertical mode, only one N atom in the N_2_ molecule interacts with the metal leading to weak activation (Song et al., 2021).

The dissociated adsorption energy of the N_2_ molecule on the catalytic systems is calculated as follows:

$${\text{E}}_{\text{ad}}{\;=\text{ E}}_{\left(\text{N}2----\text{M}--\text{system}\right)}-{\text{E}}_{\left(\text{N}2\right)}-{\text{E}}_{\left(\text{M}--\text{system}\right)}\text{,}$$

where E_(N2----M--system)_ represents the energy of the dissociated N_2_ molecule on the catalytic systems. E_(N2)_ and E_(M--system)_ represent the energy of the N_2_ molecule and metal-adsorbed various surface supports, respectively. Nudged elastic band (NEB) calculations were performed toward prediction of energy barrier of N_2_ activation on metal-adsorbed BN-doped graphene-based substrates.

## Results and Discussion

### Anchoring of Single Metal Atom (Mo, Fe, and V) on Various Graphene-Based Supports

**FIGURE 1:**
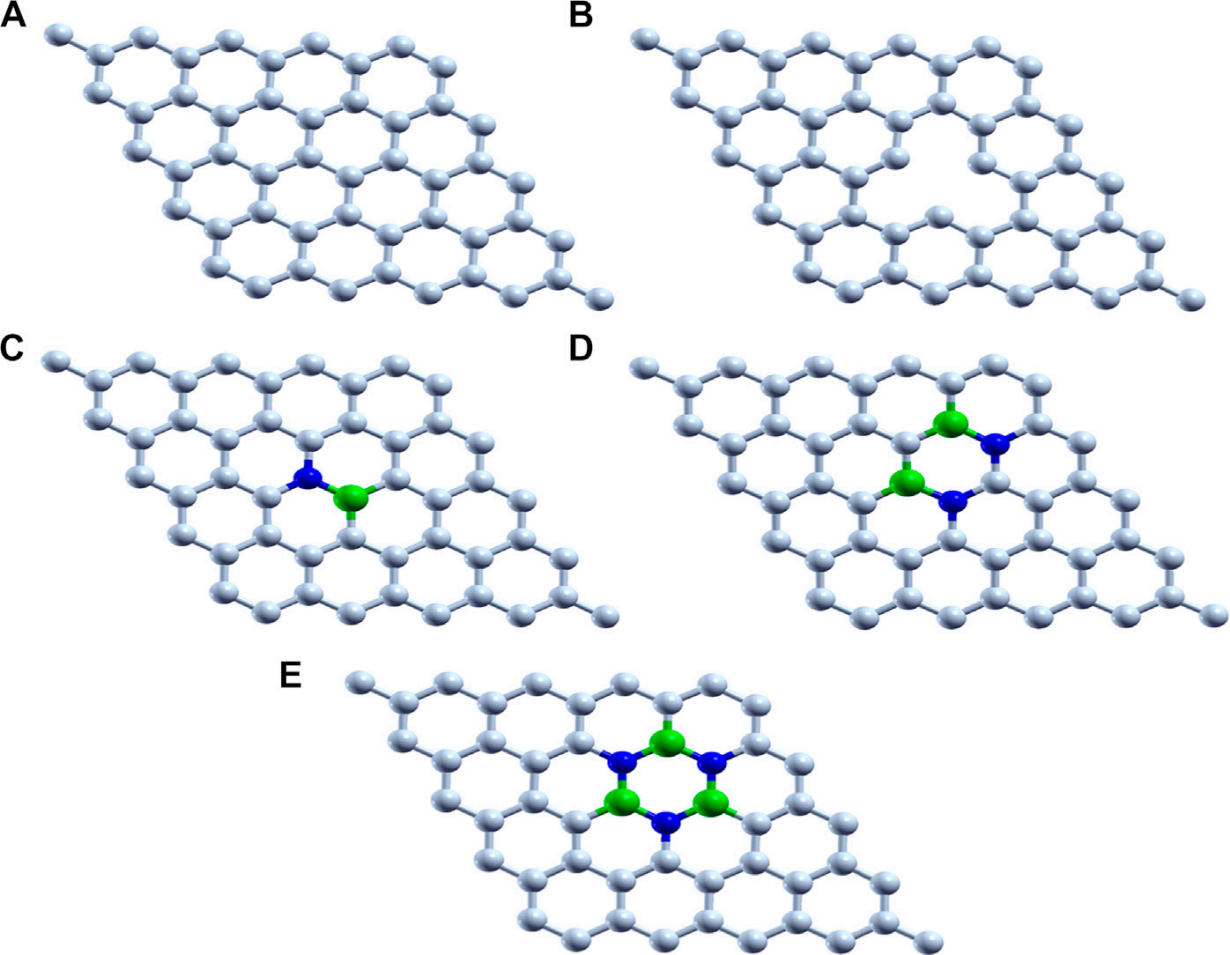
Optimized structure of **(A)** pristine graphene, **(B)** defective graphene, **(C)** BN-doped graphene, **(D)** BNC-ring graphene, and **(E)** BN-ring graphene (C, white; B, green; and N, blue).

Graphene-based 2D materials which act as an electron reservoir are used as the support for adsorbing the single atom cluster (Mo, Fe, and V) which increases the catalytic activity of the metal center. The five graphene-based supports are designed, viz., 1) pristine graphene (50 carbon atoms), 2) defective graphene (49 carbon atoms with a single vacancy at the center), 3) BN-doped graphene (4% heteroatom doping in which boron and nitrogen are substituted instead of carbon in the pristine graphene), 4) BNC-ring graphene (8% heteroatom doping), and 5) BN-ring graphene (12% heteroatom doping). All these graphene-based supports are designed and optimized to the local minima as shown in Figure 1.

**FIGURE 2:**
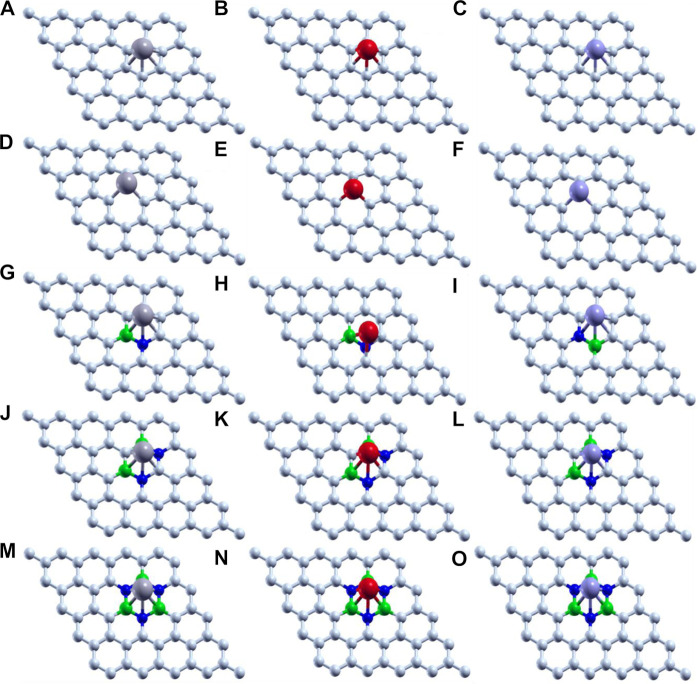
Optimized structure of adsorption of Mo (gray), Fe (red), and V (purple) on **(A–C)** pristine graphene, **(D–F)** defective graphene, **(G–I)** BN-doped graphene, **(J–L)** BNC-ring graphene, and **(M–O)** BN-ring graphene.

Thus, we have tried to establish the relative reactivity of single atom clusters (Mo, Fe, and V) chemisorbed on the above-mentioned surfaces. The optimized structure of adsorption of Mo (gray), Fe (red), and V (purple) on various surface supports is shown in Figure 2. The adsorption energy of a single metal atom (Mo, Fe, and V) on pristine graphene, defective graphene, BN-doped graphene, BNC-ring graphene, and BN-ring graphene is 4.653, 2.602, and 3.145 eV; 10.893, 9.329, and 9.744 eV; 3.929, 1.090, and 2.494 eV; 3.864, 1.728, and 2.498 eV; and 3.016, 1.048, and 1.467 eV, respectively. Comparatively, the adsorption energy of Mo on the designed supports is ∼2 eV more due to its bulky nature with respect to other metals (Fe and V). Interestingly, the dangling carbon atoms at the center increase the adsorption energies for a defective graphene support better than the rest, and also the increase in the percentage of heteroatom (B and N) doping decreases the adsorption energies of the single metal atom on supports.

**FIGURE 3:**
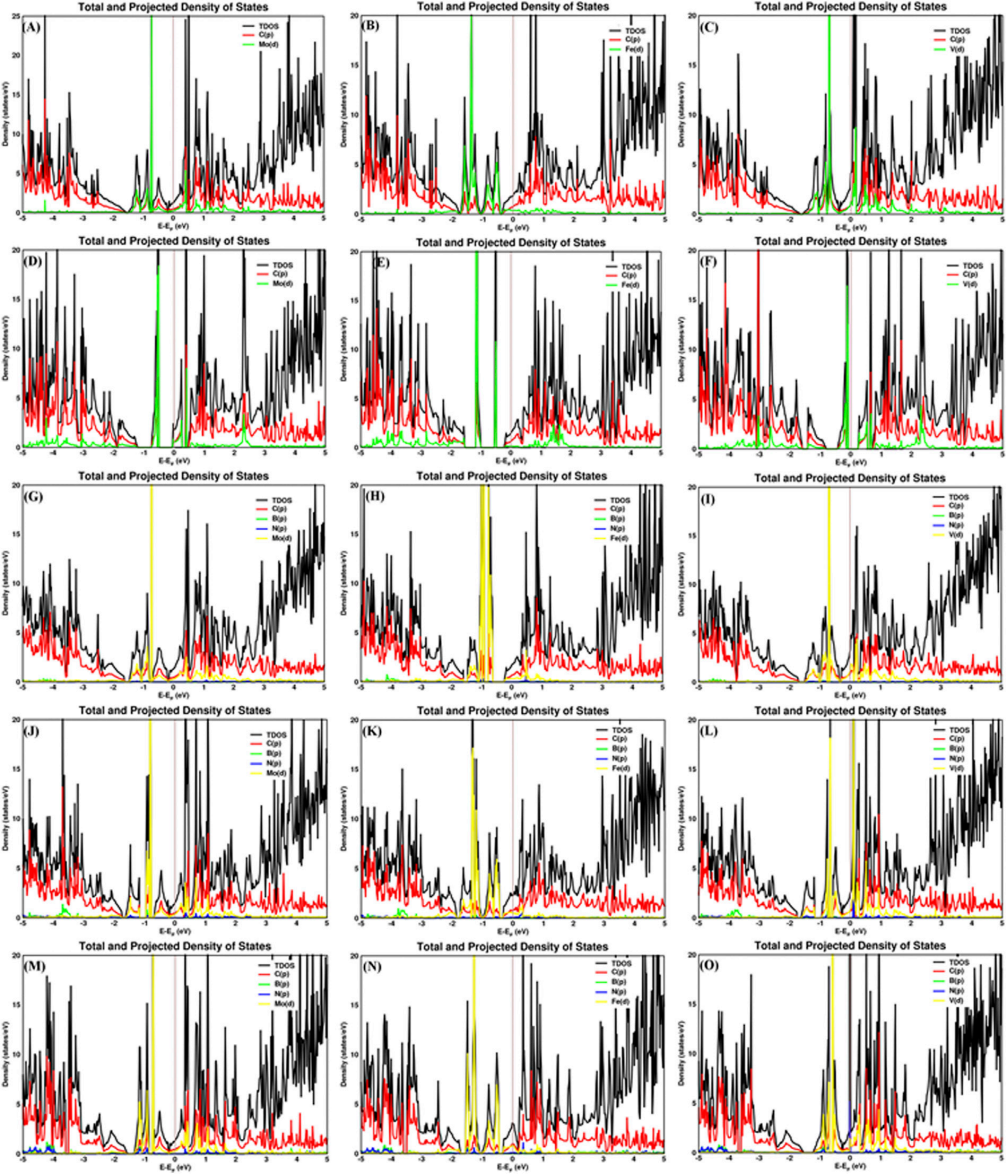
Total and projected density of states of Mo, Fe, and V on **(A–C)** pristine graphene, **(D–F)** defective graphene, **(G–I)** BN-doped graphene, **(J–L)** BNC-ring graphene, and **(M–O)** BN-ring graphene.

The carbon–metal (C–M) interatomic distance of Mo, Fe, and V on pristine graphene, defective graphene, BN-doped graphene, and BNC-ring graphene is 2.200–2.213, 2.069–2.080, and 2.147–2.170 Å;1.932–1.956, 1.766–1.768, and 1.863–1.873 Å; 2.145–2.261, 2.012–2.473, and 2.079–2.192 Å; and 2.072–2.305, 1.947–2.148, and 2.072–2.306 Å, respectively. The boron–metal (B–M) interatomic distance of Mo, Fe, and V on BN-doped graphene, BNC-ring graphene, and BN-ring graphene is 2.258, 2.303, and 2.215 Å; 2.279–2.28, 2.106–2.218, and 2.279–2.28 Å; and 2.216–2.219, 2.063–2.124, and 2.17–2.227 Å, respectively. The nitrogen–metal (N–M) interatomic distance of Mo, Fe, and V on BN-doped graphene, BNC-ring graphene, and BN-ring graphene is 2.211, 1.861, and 2.172 Å; 2.224–2.226, 2.01–2.225, and 2.224–2.226 Å; and 2.267–2.273, 2.073–2.197, and 2.204–2.226 Å, respectively. The interatomic distances and adsorption energies of Mo, Fe, and V on various graphene-based supports are shown in Table 1. Thus, the significance of the result shows that the adsorption energies of a single metal atom on the surface support provide a stable and potential catalyst for N_2_ activation. The total density of states and projected density of states of a single metal atom (Mo, Fe, and V) on graphene-based supports are shown in Figure 3. The total density of states (TDOS) and partial density of states (PDOS) reveal that the d-states of a single metal atom (Mo, Fe, and V) strongly hybridize with the p-state of unsaturated carbon atoms and heteroatoms (B and N). The d-state of a single metal atom shows its maximum density of states between −2 and 2 eV. On comparing, the p-state of unsaturated carbon atoms is maximum in pristine and defective supports which reveals that, in the other three supports, the p-state of both boron and nitrogen is hybridized with the d-state of metal.

**TABLE 1:** Interatomic distances and adsorption energies of Mo, Fe, and V on various graphene-based supports (pristine graphene, defective graphene, BN-doped graphene, BNC-ring graphene, BN-ring graphene, and adsorption energy are abbreviated as prisgp, dfgp, BNgp, BNCgp, BNrg_gp, and E_ad_).

System	C–metal (Å)	B–metal (Å)	N–metal (Å)	E_ad_ (eV)
prisgp_Mo	2.200–2.213	—	—	−4.653
prisgp_Fe	2.069–2.080	—	—	−2.602
prisgp_V	2.147–2.170	—	—	−3.145
dfgp_Mo	1.932–1.956	—	—	−10.893
dfgp_Fe	1.766–1.768	—	—	−9.329
dfgp_V	1.863–1.873			−9.744
BNgp_Mo	2.145–2.261	2.258	2.211	−3.929
BNgp_Fe	2.012–2.473	2.303	1.861	−1.090
BNgp_V	2.079–2.192	2.215	2.172	−2.494
BNCgp_Mo	2.072–2.305	2.279–2.28	2.224–2.226	−3.864
BNCgp_Fe	1.947–2.148	2.106–2.218	2.01–2.225	−1.728
BNCgp_V	2.072–2.306	2.279–2.28	2.224–2.226	−2.498
BNrg_gp_Mo	—	2.216–2.219	2.267–2.273	−3.016
BNrg_gp_Fe	—	2.063–2.124	2.073–2.197	−1.048
BNrg_gp_V	—	2.17–2.227	2.204–2.226	−1.467

### N_2_ Activation on Single Metal Atom (Mo, Fe, and V) Anchored on Various Graphene-Based Supports

**FIGURE 4:**
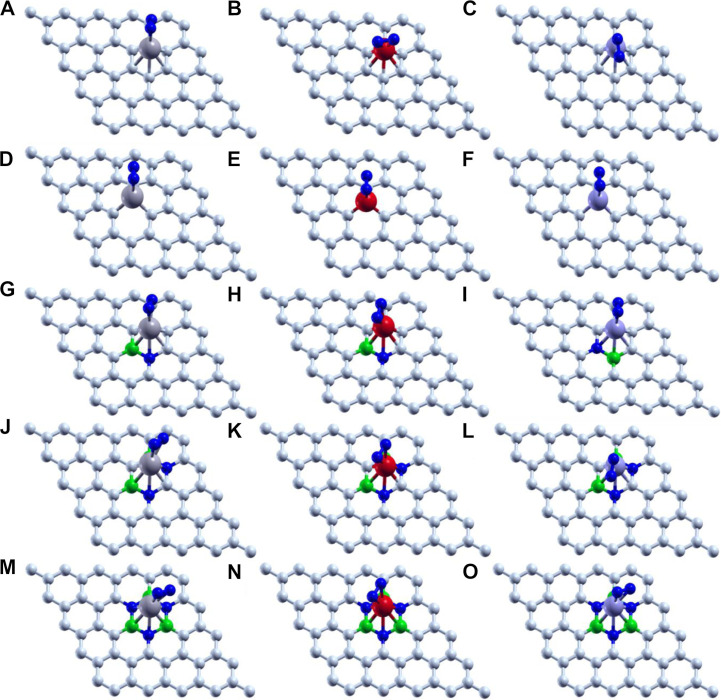
Optimized structure of the N_2_ molecule adsorbed on Mo (gray), Fe (red), and V (purple) on various supports: **(A–C)** pristine graphene, **(D–F)** defective graphene, **(G–I)** BN-doped graphene, **(J–L)** BNC-ring graphene, and **(M–O)** BN-ring graphene.

The adsorption energies of N_2_ on a single metal atom (Mo, Fe, and V) on pristine graphene, defective graphene, BN-doped graphene, BNC-ring graphene, and BN-ring graphene are 1.739, 1.334, and 1.996 eV; 0.887, 0.620, and 0.628 eV; 1.844, 2.278, and 1.988 eV; 1.870, 1.544, and 2.116 eV; and 1.868, 1.510, and 2.258 eV, respectively. Comparatively, the adsorption energies of N_2_ on a single metal atom (Mo, Fe, and V) on the defective graphene support are less compared to those on the rest of the support. Moreover, there is an eventual increase in adsorption energies of N_2_ on V on supports (BN-doped graphene, BNC-ring graphene, and BN-ring graphene) due to more vacant *d* orbitals (less than half-filled), which is vice versa in Fe (more than half-filled *d* orbitals) on the same supports. The optimized structure of adsorption of N_2_ on a single metal atom (Mo, Fe, and V) on various surface supports is shown in Figure 4.

The carbon–metal (C–M) interatomic distance of N_2_ on a single metal atom (Mo, Fe, and V) on pristine graphene, defective graphene, BN-doped graphene, and BNC-ring graphene is 2.228–2.313, 2.077–2.162, and 2.190–2.264 Å; 1.946–2.013, 1.777–1.847, and 1.871–1.925 Å; 2.194–2.257, 2.096–2.144, and 2.133–2.234 Å; and 2.111–2.319, 2.004–2.172, and 2.048–2.241 Å, respectively. The boron–metal (B–M) interatomic distance of N_2_ on a single metal atom (Mo, Fe, and V) on BN-doped graphene, BNC-ring graphene, and BN-ring graphene is 2.313, 2.18, and 2.319 Å; 2.316–2.407, 2.219–2.222, and 2.303–2.342 Å; and 2.244–2.41, 2.141–2.216, and 2.256–2.371 Å, respectively. The nitrogen–metal (N_doped_–M) interatomic distance of N_2_ on a single metal atom (Mo, Fe, and V) on BN-doped graphene, BNC-ring graphene, and BN-ring graphene is 2.32, 2.202, and 2.223 Å; 2.239–2.328, 2.165–2.173, and 2.23–2.242 Å; and 2.274–2.341, 2.083–2.234, and 2.264–2.297 Å, respectively.

**FIGURE 5:**
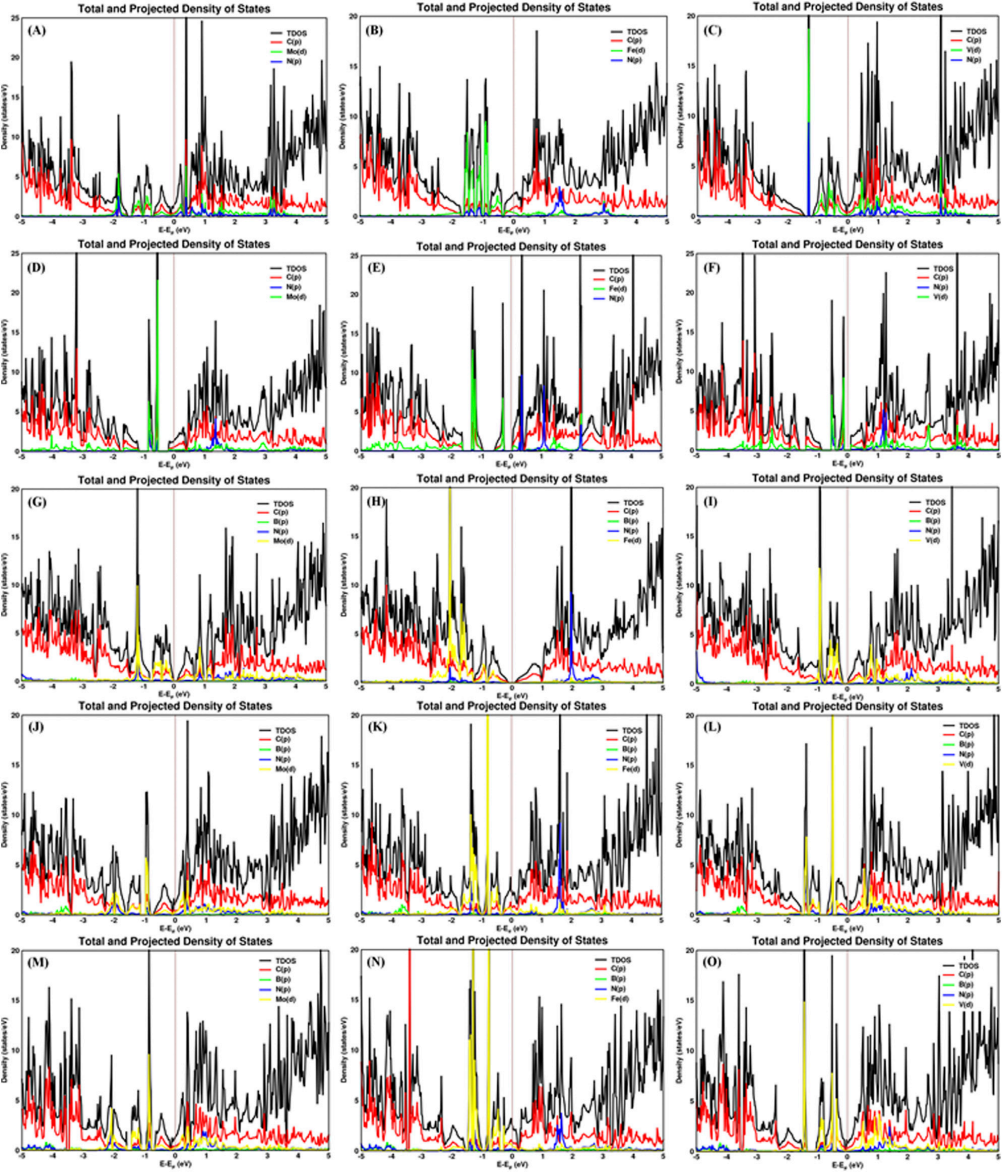
Total and projected density of states of the N_2_ molecule adsorbed on Mo, Fe, and V on various supports: **(A–C)** pristine graphene, **(D–F)** defective graphene, **(G–I)** BN-doped graphene, **(J–L)** BNC-ring graphene, and **(M–O)** BN-ring graphene.

The nitrogen–metal (N_ad_–M) interatomic distance of N_2_ on a single metal atom (Mo, Fe, and V) on pristine graphene, defective graphene, BN-doped graphene, BNC-ring graphene, and BN-ring graphene is 2.039–2.117, 1.92–1.923, and 1.911–1.994 Å; 2.22–2.221, 1.964–2.078, and 2.161–2.218 Å; 2.027–2.091, 1.902–1.907, and 1.918–2 Å; 2–2.057, 1.889–1.89, and 1.908–1.979 Å; and 2–2.068, 1.9, and 1.869–1.928 Å, respectively. The interatomic distances and adsorption energy of N_2_ on a single metal atom (Mo, Fe, and V) on various substrate systems are shown in Table 2. The total density of states and projected density of states of N_2_ on a single metal atom (Mo, Fe, and V) on the graphene-based support are shown in Figure 5. The total density of states (TDOS) and partial density of states (PDOS) reveal that the d-states of a single metal atom (Mo, Fe, and V) hybridize with the p-state of adsorbed nitrogen as well as carbon, boron, and nitrogen atoms doped on the support. Thus, the d-state of a single metal atom shares its vacant orbital with the p-state of hybridizing atoms.

**TABLE 2:** Interatomic distances and adsorption energies of N_2_ on Mo, V, and Fe on various graphene-based supports (pristine graphene, defective graphene, BN-doped graphene, BNC-ring graphene, BN-ring graphene, and adsorption energy are abbreviated as prisgp, dfgp, BNgp, BNCgp, BNrg_gp, and E_ad_).

System	C–metal (Å)	B–metal (Å)	N_doped_–metal (Å)	N_ad_–metal (Å)	E_ad_ (eV)

prisgp_Mo	2.228–2.313	—	—	2.039–2.117	−1.739
prisgp_Fe	2.077–2.162	—	—	1.92–1.923	−1.334
prisgp_V	2.190–2.264	—	—	1.911–1.994	−1.996
dfgp_Mo	1.946–2.013	—	—	2.22–2.221	−0.887
dfgp_Fe	1.777–1.847	—	—	1.964–2.078	−0.620
dfgp_V	1.871–1.925	—	—	2.161–2.218	−0.628
BNgp_Mo	2.194–2.257	2.313	2.32	2.027–2.091	−1.844
BNgp_Fe	2.096–2.144	2.18	2.202	1.902–1.907	−2.278
BNgp_V	2.133–2.234	2.319	2.223	1.918–2	−1.988
BNCgp_Mo	2.111–2.319	2.316–2.407	2.239–2.328	2–2.057	−1.870
BNCgp_Fe	2.004–2.172	2.219–2.222	2.165–2.173	1.889–1.89	−1.544
BNCgp_V	2.048–2.241	2.303–2.342	2.23–2.242	1.908–1.979	−2.116
BNrg_gp_Mo	—	2.244–2.41	2.274–2.341	2–2.068	−1.868
BNrg_gp_Fe	—	2.141–2.216	2.083–2.234	1.9	−1.510
BNrg_gp_V	—	2.256–2.371	2.264–2.297	1.869–1.928	−2.258

### N–N Bond Stretching Frequency Analysis on Designed Catalytic Systems

To probe the stretching frequency of the adsorbed N_2_ molecule on a single metal atom (Mo, Fe, and V) on the graphene-based support, we investigated the spectral range of 1,300–2,300 cm^−1^, which covers the typical frequencies of the different N_2_ species known to exist on transition-metal surfaces. The stretching frequency of the unbound N_2_ molecule is attributed to 2,330 cm^−1^, and the N–N bond length is 1.09 Å (Shi and Jacobi, 1992). The N–N bond length and IR stretching frequency ν(N–N) of N_2_ on a single metal atom (Mo, Fe, and V) on pristine graphene, defective graphene, BN-doped graphene, BNC-ring graphene, and BN-ring graphene are 1.20 Å (1735 cm^−1^), 1.18 Å (1823 cm^−1^), and 1.21 Å (1,692 cm^−1^); 1.17 Å (1907 cm^−1^), 1.16 Å (2009 cm^−1^), and 1.16 Å (1997 cm^−1^); 1.20 Å (1701 cm^−1^), 1.19 Å (1802 cm^−1^), and 1.20 Å (1711 cm^−1^); 1.21 Å (1,636 cm^−1^), 1.19 Å (1777 cm^−1^), and 1.21 Å (1,688 cm^−1^); and 1.21 Å (1,666 cm^−1^), 1.19 Å (1796 cm^−1^), and 1.23 Å (1,597 cm^−1^), respectively.

The Bader charge analysis (Bader, 1991; Tang et al., 2009) clearly demonstrates the charge redistribution between the activated nitrogen atoms and the active metal centered on support-based catalysts. The structural, electronic, and vibrational properties of various catalytic systems for N_2_ activation are listed in Table 3. The N–N stretching frequency, N–N bond length, and charge on nitrogen of the N_2_ molecule adsorbed on Mo, Fe, and V on various graphene supports are shown in Supplementary Figure S1.

**TABLE 3:** Structural, electronic, and vibrational properties of various catalytic systems for N_2_ activation (pristine graphene, defective graphene, BN-doped graphene, BNC-ring graphene, and BN-ring graphene are abbreviated as prisgp, dfgp, BNgp, BNCgp, and BNrg_gp).

System	N–N bond length	IR stretching	Charge on N_2_ (e)	
	(Å)	(cm^−1^)		
prisgp_Mo	1.2	1735	−0.3239	−0.2274
prisgp_Fe	1.18	1823	−0.1556	−0.3095
prisgp_V	1.21	1,692	−0.4328	−0.1847
dfgp_Mo	1.17	1907	−0.3055	−0.1237
dfgp_Fe	1.16	2009	−0.1954	−0.1228
dfgp_V	1.16	1997	−0.1359	−0.1935
BNgp_Mo	1.2	1701	−0.2088	−0.363
BNgp_Fe	1.19	1802	−0.2152	−0.252
BNgp_V	1.2	1711	−0.2733	−0.3433
BNCgp_Mo	1.21	1,636	−0.2783	−0.3213
BNCgp_Fe	1.19	1777	−0.2714	−0.2093
BNCgp_V	1.21	1,688	−0.2711	−0.352
BNrg_gp_Mo	1.21	1,666	−0.2584	−0.3194
BNrg_gp_Fe	1.19	1796	−0.314	−0.1728
BNrg_gp_V	1.23	1,597	−0.2666	−0.4262

As a case study, the potential of the Mo-adsorbed BN-doped graphene catalyst for the activation of N_2_ is discussed in Supplementary Figure S2. NEB calculation is performed in between these reactants and products to confirm the N_2_ activation energy barrier. Mo-adsorbed BN-doped graphene and gaseous nitrogen are considered reactants. Thus, the Mo-adsorbed BN-doped graphene catalyst shows more feasible N_2_ activation with an effective energy barrier of 3.21 eV. The activation barrier plot of the N_2_ molecule adsorbed on Mo on the BN-doped graphene support is shown in Supplementary Figure S2 (Liu et al., 2021).

## Conclusion

In this work, we explore the potential of various graphene-based 2D materials, viz., pristine, defective, BN-doped graphene, etc., as a support for a single atom cluster (Mo, Fe, and V). These graphene-based supports show excellent potential toward the anchoring of a single atom cluster (Mo, Fe, and V) with adsorption energies ranging between 1.048 and 10.893 eV. Thus, the adsorption energies vary substantially with respect to the graphene-based supports, viz., pristine, defective, BN doped, etc. This is attributed to the size and nature of hybridization between the *d* orbitals of the interacting single metal atom (Mo, Fe, and V) and the *sp* ^*2*^ orbitals of unsaturated carbon atoms of various designed graphene-based supports. The catalytic performance of a single metal atom (Mo, Fe, and V) on graphene-supported catalysts is explored for the activation of molecular nitrogen. The adsorption energies of the nitrogen molecule on a graphene-supported single atom cluster (Mo, Fe, and V) range between 0.620 and 2.278 eV, which is attributed to the interacting environment of the active metal centered on the support and the *p* orbital of adsorbed molecular nitrogen. Bader charge and density of states analyses corroborate an enhanced hybridization between the *d* states of the single metal atoms (Mo, Fe, and V) and adsorbed molecular nitrogen for activation. The N–N stretching frequencies are found which are considerably red-shifted ranging from 2009 cm^−1^ (1.16 Å) to 1,597 cm^−1^ (1.23 Å) compared to that of the unbound N_2_ molecule (2,330 cm^−1^ (1.09 Å)). Thus, from the results, we understood that even a single metal atom (Mo, Fe, and V) with functionalized (BN-doped) graphene supports can highlight the excellent potential for nitrogen activation.

## Data Availability

The original contributions presented in the study are included in the article/Supplementary Material, and further inquiries can be directed to the corresponding authors.
