# Supplementary material for: Role of Chemical Structure of Support in Enhancing the Catalytic Activity of a Single Atom Catalyst Toward NRR: A Computational Study
Source: Front Chem. 2021 Sep 8;9:733422. doi: 10.3389/fchem.2021.733422 (PMC8455884; doi:10.3389/fchem.2021.733422)
Supplement: Supplementary file 1 [file DataSheet1.docx]

**Supporting Information**

**Role of chemical structure of support in enhancing the catalytic activity of a Single atom catalyst towards NRR. A Computational Study**

Thillai Govindaraja Senthamaraikannan*,^a,b**^* Selvaraj Kaliaperumal*^b^* and Sailaja Krishnamurty*^c*^*

*^a^Department of Environmental Engineering, Chungbuk National University,*

*Chungdae-ro 1, Seowon-gu, Cheongju, Chungbuk 28644, Republic of Korea*

*^b^Nano and Computational Material Lab., Catalysis Division, CSIR-National Chemical Laboratory, Pune 411 008, India*

*^c^Physical Chemistry Division, CSIR-National Chemical Laboratory, Pune 411 008, India*

*Corresponding author: Email: [k.sailaja@ncl.res.in](mailto:k.sailaja@ncl.res.in?subject=)

**Co-Corresponding author: Email: thillaincl@gmail.com


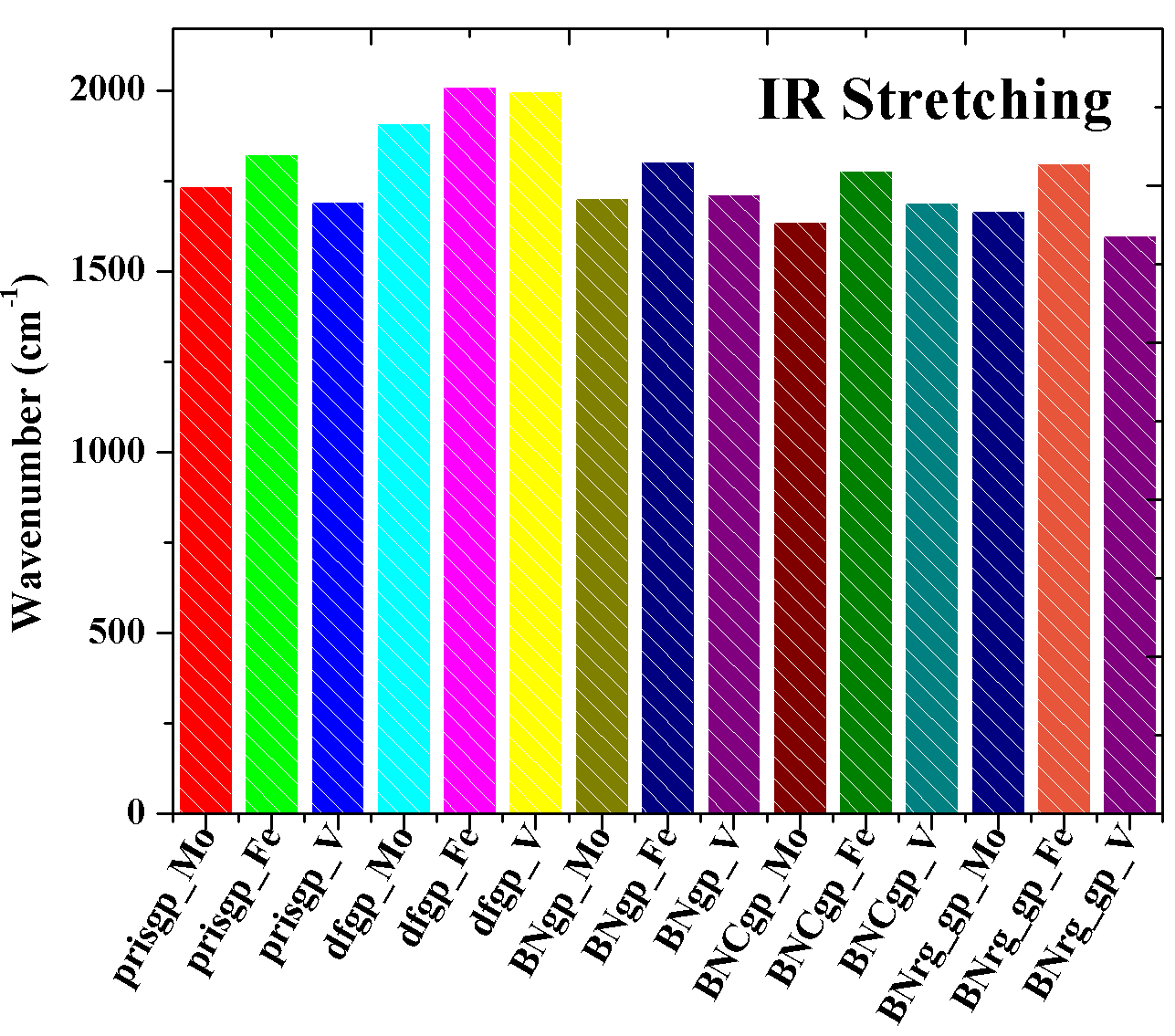

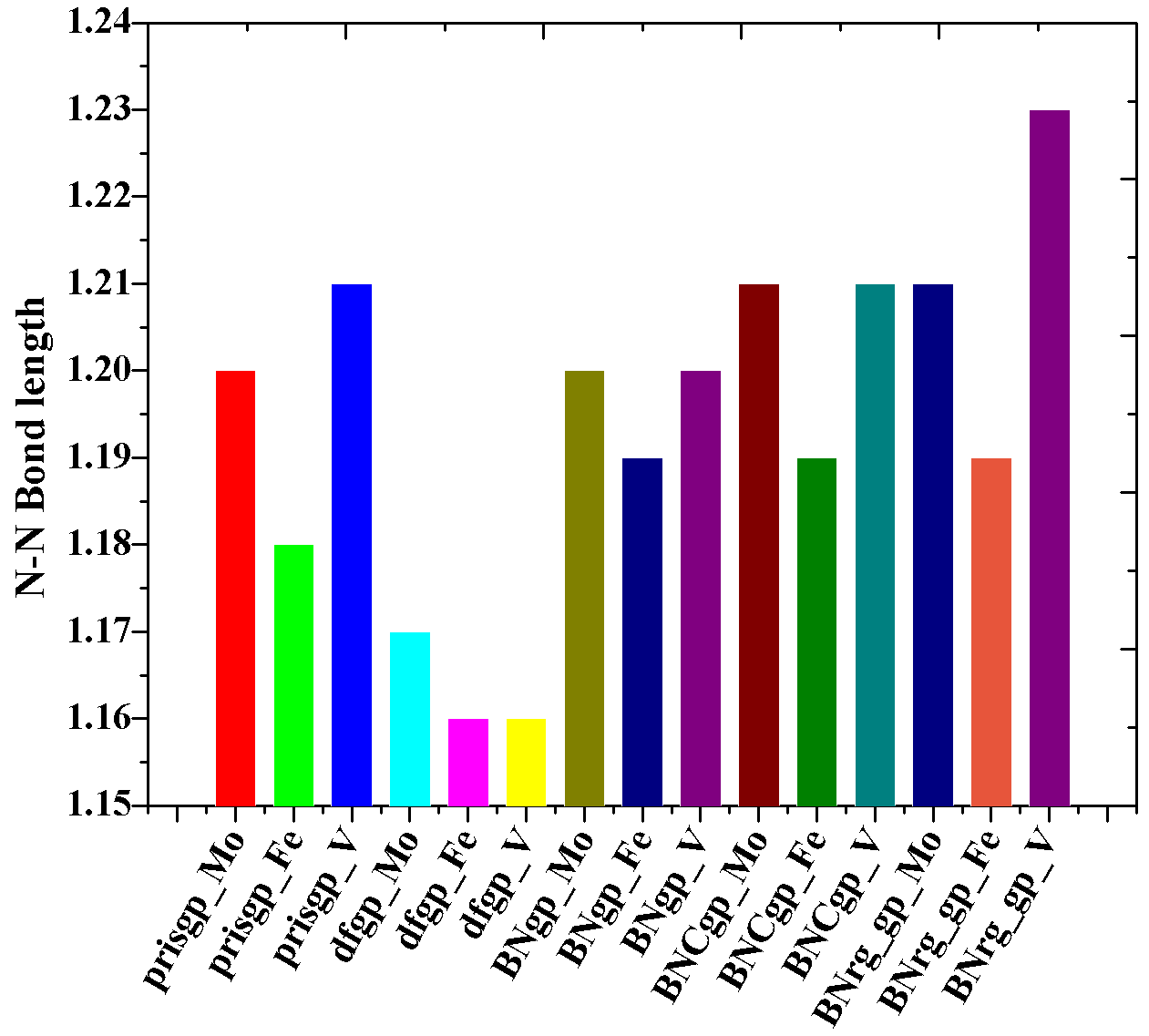

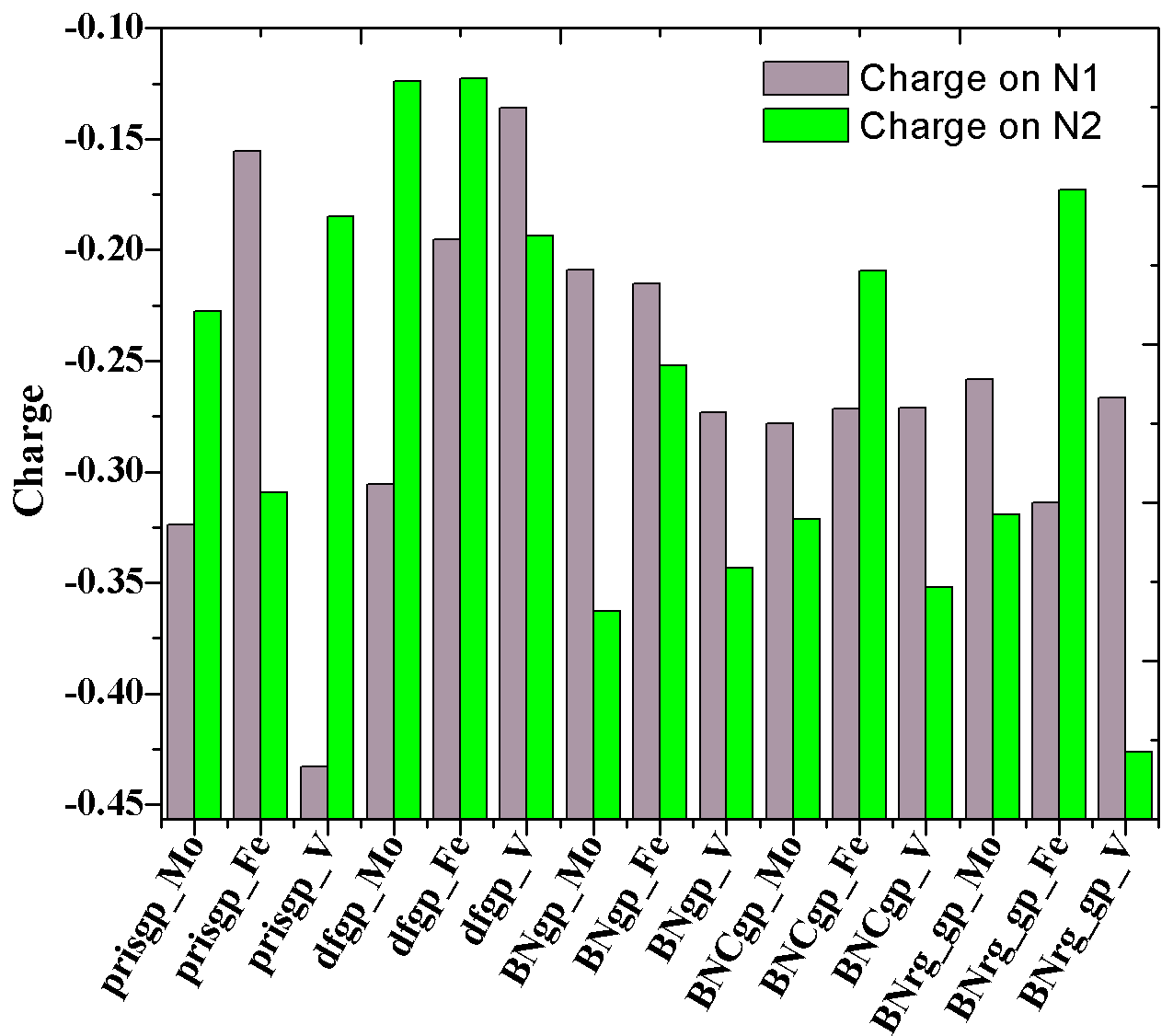


**(b)**

**(a)**

**(c)**

Figure S1. The (a) IR stretching frequency, (b) N-N bond length and (c) Charge on nitrogen of N_2_ molecule adsorped on Mo, Fe and V on various graphene support


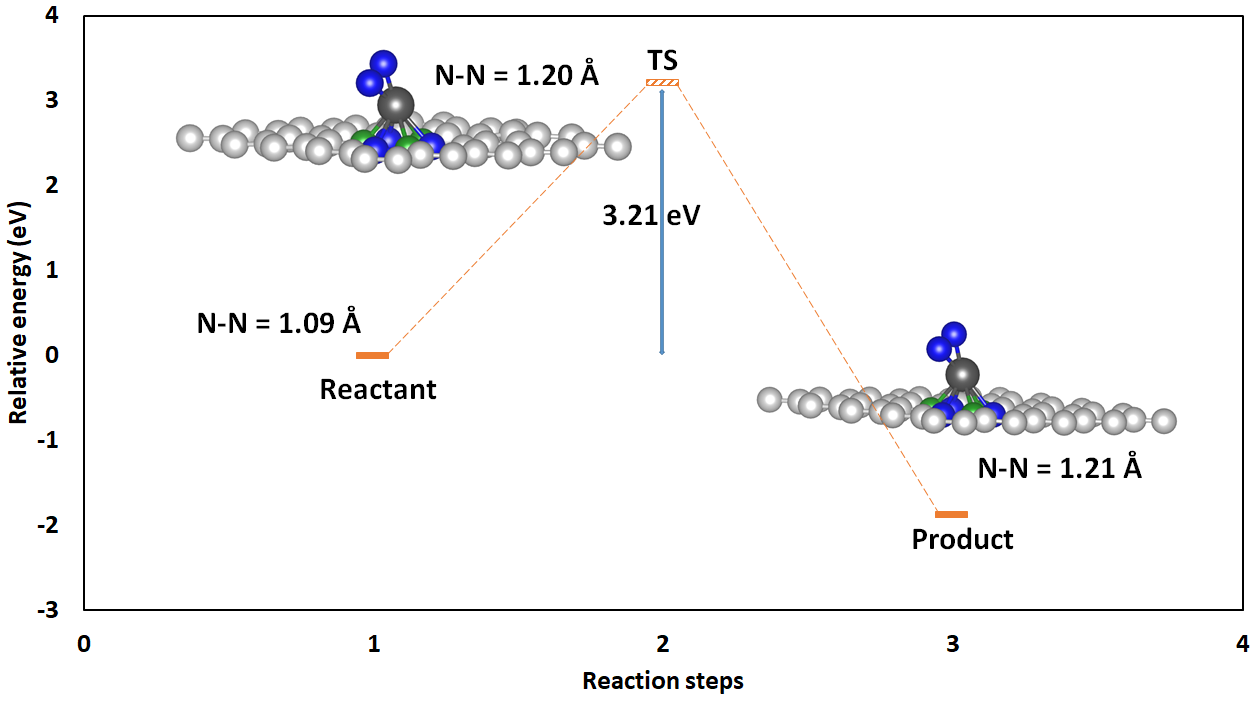


Figure S2. The activation barrier plot of N_2_ molecule adsorbed on Mo on BN doped graphene support.

As case study, the potential of Mo adsorbed BN-doped graphene catalyst for the activation of N_2_ has been discussed in Figure S2. NEB calculation is performed in between these reactant and product to confirm the N_2_ activation energy barrier. Mo adsorbed BN-doped graphene and gaseous nitrogen are considered as reactant. Thus, Mo adsorbed BN-doped graphene catalyst shows more feasible N_2_ activation with an energy barrier of 3.21 eV.
